# Supplementary material for: Quantum corrections to the magnetoconductivity of surface states in three-dimensional topological insulators
Source: Nat Commun. 2023 May 5;14:2596. doi: 10.1038/s41467-023-38256-4 (PMC10163031; doi:10.1038/s41467-023-38256-4)
Supplement: Supplementary file 1 — Supplementary Information [file 41467_2023_38256_MOESM1_ESM.pdf]

Supplementary Information to

Quantum corrections to the magnetoconductivity of surface states in three-dimensional topological insulators

Gang Shi,[1,2] Fan Gao,[1,2] Zhilin Li,[1,3] Rencong Zhang,[1,2] Igor Gornyi,[4,5,6] Dmitri Gutman,[7,*] and Yongqing Li[1,2,3,†]

[1]*Beijing National Laboratory for Condensed Matter Physics, Institute of Physics, Chinese Academy of Sciences, Beijing 100190, China*

[2]*School of Physical Sciences, University of Chinese Academy of Sciences, Beijing 100049, China*

[3]*Songshan Lake Materials Laboratory, Dongguan, Guangdong 523808, China*

[4]*Institute for Quantum Materials and Technologies, Karlsruhe Institute of Technology, 76021 Karlsruhe, Germany*

[5]*Institut für Theorie der Kondensierten Materie, Karlsruhe Institute of Technology, 76128 Karlsruhe, Germany*

[6]*Ioffe Institute, 194021 St. Petersburg, Russia*

[7]*Department of Physics, Bar-Ilan University, Ramat Gan, 52900, Israel*

I. SUPPLEMENTARY NOTE 1

Theory of quantum corrections to magnetoconductance in 2D systems of symplectic symmetry

The surface state of topological insulators (TIs) is a metallic state with strong spin-orbit interaction. From the symmetry point of view, this disordered metal belongs to the symplectic symmetry class. The topological protection is associated with the time-reversal symmetry $\mathcal{T}$ that squares to minus one: $\mathcal{T}^2 = -1$. The external magnetic field breaks the time-reversal symmetry, driving the system to the unitary class. The magnetoresistance of the topological insulator (TI) can therefore be understood as a crossover between the symplectic and the unitary ensembles. The one-loop description of such a transition was derived in Ref.[S1]. The computation performed by Hikami, Larkin, and Nagaoka (HLN) took into account the first-order correction to the conductance in $1/g$, the inverse dimensionless conductance. For metals with moderate values of dimensionless conductance, the subleading terms play a substantial role. Here we show how to extend the HLN result to account for the next subleading term. We first show how this in the context of non-topological metals in the symplectic class and then we apply the same ideas for TIs.

In the absence of interactions, the conductance of a disordered metal is governed by one-parameter scaling theory[S1]. It can be described by the renormalization group (RG) equation:

$$-\frac{dt}{d\ln(\mathcal{L}/l_{\rm tr})} = \beta(t)\,, \tag{S1}$$

where t is the dimensionless coupling constant, inversely proportional to g. The RG flow occurs with a running scale $\mathcal{L}$ which is cut off by the physical spatial scale restricting the quantum interference. The diffusion motion of electrons starts at the transport scattering length $l_{\rm tr}$ that serves as the microscopic scale for the theory.

A. Magnetoconductance in non-topological symplectic metals

We consider a two-dimensional disordered metallic film formed by spinful electrons. On the level of Drude formula, the conductance (summed over both spin components) is given by

$$\sigma = \frac{e^2}{h} g^{(0)}. \tag{S2}$$

Here we have introduced the bare value of dimensionless conductance

$$g^{(0)} = \frac{p_F l_{\rm tr}}{\hbar} = k_F l_{\rm tr}\,, \tag{S3}$$

where $l_{\rm tr} = v_F \tau_{\rm tr}$ with $\tau_{\rm tr}$ the transport scattering time and v_F the Fermi velocity. The Fermi momentum p_F is related to the total density of electrons n by the standard formula (both spins included, hence an overall factor of 2)

$$n = 2\int_{|p|<p_F} \frac{d^2p}{(2\pi\hbar)^2} = \frac{p_F^2}{2\pi\hbar^2}\,. \tag{S4}$$

To account for the localization effects, one uses the one-parameter scaling theory[2], Eq. (S1). The β-function is determined by the symmetry class, controlled by the ratio between several length scales. The magnetic field suppresses quantum interference for distances greater than magnetic length $l_{\rm B} = \sqrt{\hbar/(eB)}$. The effects of spin-orbit are characterized by $l_{\rm so}$. The coherence in the sample is destroyed by dephasing at length l_φ. The RG flow terminates by the sample size $L_{\rm sample}$ when it is smaller than l_φ.

Within the two-loop approximation, the β-function reads[S2,S3,S4]

$$\beta(t) \simeq \begin{cases} -2t^2, & \text{orthogonal,} \\ -2t^3, & \text{unitary,} \\ t^2, & \text{symplectic.} \end{cases} \tag{S5}$$

Here

$$t = \frac{1}{\pi g}, \tag{S6}$$

is the dimensionless resistance, where g is the total dimensionless conductance of spinful electrons.

For the unitary case, the one-loop localization correction is absent. Therefore, the MC starts with terms proportional to $1/g$:

$$g(\mathcal{L}) = g^{(0)} - \frac{1+3}{2\pi^2 g^{(0)}} \ln\frac{\mathcal{L}}{l_{\rm tr}}\,. \tag{S7}$$

Here we have separated the contributions to the logarithmic correction coming from the singlet channel (with the coefficient 1) and from the triplet channels (with the coefficient 3). Since in the unitary class the Cooperons are suppressed by the magnetic field, the two-loop correction stems solely from diagrams with the diffuson modes.

For the orthogonal case, the two-loop correction vanishes. This implies that the two-loop contributions coming from the diffuson modes are canceled by the contributions involving Cooperons. Because we know the value of the diffuson contribution from the unitary-class calculation, Eq. (S7), we can now describe the crossover between the orthogonal and unitary ensembles[S5], by adding and subtracting the unitary-class term to the one-loop orthogonal-class correction:

$$g(\mathcal{L}) = g^{(0)} + \frac{1-3}{\pi}\ln\frac{\mathcal{L}}{l_{\rm tr}} - \frac{1+3}{2\pi^2 g^{(0)}}\ln\frac{\mathcal{L}}{l_{\rm tr}} + \frac{1+3}{2\pi^2 g^{(0)}}\ln\frac{\mathcal{L}}{l_{\rm tr}}. \tag{S8}$$

Here, the first and the last logarithmic terms on the right-hand side are associated with Cooperons and hence are suppressed in the strong magnetic field, leading then to Eq. (S7). Moreover, from this expression, one can infer the results for the symplectic class by suppressing the contributions associated with the triplet channel [terms with the coefficient 3 in Eq. (S8)]:

$$g(\mathcal{L}) = g^{(0)} + \frac{1}{\pi}\ln\frac{\mathcal{L}}{l_{\rm tr}} - \frac{1}{2\pi^2 g^{(0)}}\ln\frac{\mathcal{L}}{l_{\rm tr}} + \frac{1}{2\pi^2 g^{(0)}}\ln\frac{\mathcal{L}}{l_{\rm tr}}\,. \tag{S9}$$

Now we can unite Eqs. (S9), (S8), and (S7). To do that, we bear in mind that to describe the crossover between the symplectic and other classes, one should cut off the logarithms emerging in the triplet channels by $l_{\rm so}$. In addition, the logarithmic corrections coming from the diagrams involving Cooperons are also cut off by a magnetic field in both the singlet and triplet channels. As opposed to this, the logarithms arising from the diffuson diagrams are insensitive to $l_{\rm B}$. Finally, all logarithmic corrections must be cut off by the dephasing length l_φ. Therefore, the crossover between all possible ensembles is accounted by

$$\begin{aligned} g(\mathcal{L}) &= g^{(0)} + \frac{1}{\pi}\left(\ln\frac{\min\{l_\varphi, l_{\rm B}\}}{l_{\rm tr}} - 3\ln\frac{\min\{l_\varphi, l_{\rm B}, l_{\rm so}\}}{l_{\rm tr}}\right) \\ &- \frac{1}{2\pi^2 g^{(0)}}\left(\ln\frac{l_\varphi}{l_{\rm tr}} + 3\ln\frac{\min\{l_\varphi, l_{\rm so}\}}{l_{\rm tr}} - \ln\frac{\min\{l_\varphi, l_{\rm B}\}}{l_{\rm tr}} - 3\ln\frac{\min\{l_\varphi, l_{\rm B}, l_{\rm so}\}}{l_{\rm tr}}\right). \end{aligned} \tag{S10}$$

To the zeroth order in the dimensionless conductance, this reproduces the HLN result. In addition, Eq. (S10) accounts for the terms that are proportional to the first order in $1/g$. In particular, for the regime $l_{\rm B} < l_\varphi$ one finds

$$g(\mathcal{L}) \simeq g^{(0)} - \frac{1}{2\pi}\left(1 + \frac{1}{2\pi g^{(0)}}\right)\ln\frac{B}{B_\varphi}. \tag{S11}$$

Casting this in the form

$$\Delta\sigma(B) = g(B) - g(0) = -\frac{\alpha}{\pi}\ln\frac{B}{B_\varphi} = \frac{2\alpha}{\pi}\ln\frac{l_{\rm B}}{l_\varphi}, \tag{S12}$$

we find the prefactor

$$\alpha = \frac{1}{2}\left(1 + \frac{1}{2\pi g}\right) \tag{S13}$$

for a two-dimensional non-topological symplectic metal. Note that this signifies that the value of α in units of $1/2$ has a systematic correction in the limit of clean metals ($g \gg 1$). This correction increases the values of α for metals in the symplectic symmetry class. We now proceed and apply the same logic for the metallic states on the surface of a time-reversal topological insulator.

B. Magnetoconductance of TI surface states

In TIs, the spin-orbit interaction is strong, leading to the "spin-momentum locking". As a result, the effective spin-orbit length is the shortest length scale, $l_{\rm so} < l_{\rm tr}$. The transport is carried by two parallel surfaces, each one hosting Dirac electrons. Therefore, it makes sense to describe the problem in terms of the one-surface conductivity σ_1. The total conductivity of the TI is a sum of the two contributions; for equivalent surfaces, we have just a factor of two:

$$\sigma_{\rm total} = 2\sigma_1.$$

The bare value of electric conductivity at one surface is given by Drude formula:

$$\sigma_1 = \frac{e^2}{h} g_1^{(0)}. \tag{S14}$$

Here the dimensionless conductance of one surface

$$g_1^{(0)} = \frac{p_F l_{\rm tr}}{2\hbar} \tag{S15}$$

Note that the bare conductivity of a single surface of TI coincides with the conductivity of one spin component of a non-topological metal.

In the absence of an external magnetic field, each surface of TI belongs to a symplectic ensemble. For the strong magnetic field, the system is driven to the unitary ensemble. Therefore, the magnetoresistance can be again described in terms of the crossover between these two ensembles. The RG equation written in terms of the coupling t remains unchanged, see Eq. (S5), but, because of the spin-momentum locking, the relation between t and conductance needs to be reformulated. Specifically, in the unitary class, the coupling constant is related to the dimensionless conductance of a single surface as

$$t = \frac{1}{2\pi g_1}\,. \tag{S16}$$

Here, the factor of 2 accounts for the contributions of the top and bottom surfaces to the total conductance. In the symplectic class, however,

$$t = \frac{1}{\pi g_1}\,. \tag{S17}$$

Solving the RG equation in the unitary case

$$\frac{dg_1}{d\ln\mathcal{L}} = -\frac{1}{2\pi^2 g_1}, \tag{S18}$$

one finds

$$g_1(\mathcal{L}) \simeq g_1^{(0)} - \frac{1}{2\pi^2 g_1^{(0)}} \ln \frac{\mathcal{L}}{l_{\rm tr}}\,. \tag{S19}$$

Note that the triplet channel here is absent and the correction is fully governed by the singlet channel. For the symplectic case the RG equation reads.

$$\frac{dg_1}{d\ln\mathcal{L}} = \frac{1}{\pi} \tag{S20}$$

The magnetoconductance (MC) of a surface is

$$g_1(\mathcal{L}) = g_1^{(0)} + \frac{1}{\pi}\ln\frac{\mathcal{L}}{l_{\rm tr}}\,. \tag{S21}$$

At finite magnetic field, and applying the same logic as we used for non-topological metals, we find a surface MC

$$g_1(\mathcal{L}) = g_1^{(0)} + \frac{1}{\pi}\ln\frac{\min\{L, l_{\rm B}\}}{l_{\rm tr}} - \frac{1}{2\pi^2 g_1^{(0)}}\ln\frac{L}{l_{\rm tr}} + \frac{1}{2\pi^2 g_1^{(0)}}\ln\frac{\min\{L, l_{\rm B}\}}{l_{\rm tr}}\,. \tag{S22}$$

In the regime $l_{\rm B} < L$ the total dimensionless conductance of the sample is given by

$$g_{\rm total}(B) = 2g_1(B) = 2g_1^{(0)} + \frac{2}{\pi}\left(1 + \frac{1}{2\pi g_1^{(0)}}\right)\ln\frac{l_{\rm B}}{l_{\rm tr}}\,. \tag{S23}$$

Casting this result as

$$g_{\rm total}(B) - g_{\rm total}(B=0) = -\frac{\alpha}{\pi}\ln\frac{B}{B_\varphi} = \frac{2\alpha}{\pi}\ln\frac{l_{\rm B}}{l_\varphi}, \tag{S24}$$

one establishes

$$\alpha = 1 + \frac{1}{2\pi g_1^{(0)}} \simeq 1 + \frac{1}{\pi g}\,. \tag{S25}$$

In fact, this value of the prefactor α can be used not only in the logarithmic dependence of the magnetoconductivity, but also in a more refined Hikami-Larkin-Nagaoka formula, which describes the crossover at $l_{\rm B} \sim l_\varphi$, as used in the main text (cf. Ref.[S5]).

C. Interaction-induced magnetoconductance

1. *Particle-hole channel*

Coulomb interaction modifies the RG flow of two-dimensional disordered Fermi liquid[S6]. In particular, this results in the logarithmically divergent corrections to the dc conductivity, known as Altshuler-Aronov (AA) effect[S7]. The

inclusion of the Cooperon diagrams into the AA effect [S5,S7]leads to the interaction-induced magnetoresistance. In the orthogonal symmetry class the problem was studied in Refs.[S5,S8]. It was shown that the combined effect of the Coulomb interaction and weak localization lead to the enhancement of conductivity at low magnetic field. In the low-temperature (relatively high B) limit ($l_T \gg l_{\rm B} \gg l_{\rm tr}$) one finds[S5]:

$$\delta\sigma_{\rm CWL} = \frac{2e^2}{\pi^2 h g^{(0)}} \ln \frac{l_{\rm B}}{l_{\rm tr}}. \tag{S26}$$

Here $l_T = \sqrt{\hbar D/k_B T}$. For weaker fields ($l_\varphi \gg l_{\rm B} \gg l_T$), the numerical prefactor in this correction changes by a factor of 3/2 [S8].

In this section, we account for analogous effects in TIs. To do that, we use the eigenfunctions of the symplectic model, which are the spinors (with $s = \pm$ describing the branch of the spectrum)

$$|\boldsymbol{k}, s\rangle = e^{i\boldsymbol{k}\cdot\boldsymbol{r}} \frac{1}{\sqrt{2}} \begin{pmatrix} 1 \\ -{\rm i}s{\rm e}^{{\rm i}\varphi_{\rm k}} \end{pmatrix}, \tag{S27}$$

parametrized by the angle of the 2D momentum $\varphi_{\boldsymbol{k}}$. For not too strong disorder, we neglect the processes that involve the states from the $-$ brach and focus entirely on the $+$ electrons.

We consider the short-range Gaussian disorder,

$$\overline{V(\boldsymbol{r})V(\boldsymbol{r}')} = V_0^2 \delta(\boldsymbol{r} - \boldsymbol{r}').$$

Here the overbar $\overline{\cdots}$ stands for averaging over disorder realizations, and V_0 quantifies the strength of the scattering potential. The scattering matrix element between the states $+, \boldsymbol{k}$ and $+, \boldsymbol{k}'$ is given by[S9]

$$\langle \boldsymbol{k}', +|V|\boldsymbol{k}, +\rangle = A_{\boldsymbol{k}'\boldsymbol{k}} V_{++} \tag{S28}$$

where $A_{\boldsymbol{k}'\boldsymbol{k}} = \int V(\boldsymbol{r}) e^{i(\boldsymbol{k}-\boldsymbol{k}')\mathbf{r}} d\boldsymbol{r}/V_0$,

$$V_{++} = V_0(1 + {\rm e}^{-{\rm i}\theta})/2,$$

and $\theta = \varphi_{\boldsymbol{k}'} - \varphi_{\boldsymbol{k}}$ is the scattering angle.

Let us compute the interaction-induced correction to MC. The diagrams in the particle-hole channel are shown in Fig. S1. These are essentially Althsuler-Aronov diagram with an insertion of the Cooperon ladder. The latter is sensitive to the external magnetic field, that induces the magneto-dependence of the conductivity.

To evaluate these diagrams, we first analyze the diffuson ladder and Cooperon ladders. The construction of these ladders involves two different probabilities W and $\tilde{W}$. The first arises if the impurity line is inserted between the

Green's functions with arrows pointing in the same direction, while the second one corresponds to the Green's functions pointing in the opposite directions:

$$W_{k,p} = V_{k,p}V_{-k,-p}\,, \quad \tilde{W}_{k,p} = V_{k,p}V_{p,k} \tag{S29}$$

Defining the angles between fast and slow momenta

$$\varphi = \varphi_{\boldsymbol{k}} - \varphi_{\boldsymbol{q}}, \qquad \varphi' = \varphi_{\boldsymbol{k}'} - \varphi_{\boldsymbol{q}}$$

one finds

$$\tilde{W}(\theta) = \frac{V_0^2}{2}(1+\cos\theta), \quad W(\theta) = e^{\mathrm{i}\theta}\tilde{W}(\theta) = \frac{V_0^2}{2}\mathrm{e}^{\mathrm{i}\theta}(1+\cos\theta). \tag{S30}$$

Considering the ladder that starts with a single disorder line, one obtains a Dyson equation for the diffuson propagator[S9]:

$$\mathcal{D}(\varphi,\varphi') = \tilde{W}(\varphi-\varphi') + \frac{2\pi\tau\nu_+}{\hbar}\left\langle \tilde{W}_+(\varphi-\varphi_1)P(\varphi_1)\mathcal{D}(\varphi_1,\varphi')\right\rangle_{\varphi_1} \tag{S31}$$

Here

$$P(\varphi) = \frac{1}{1-\mathrm{i}ql\cos\varphi + \tau/\tau_\varphi}, \qquad l = v_\mathrm{F}\tau, \tag{S32}$$

In the diffusive limit of small frequencies and momenta are then written as:

$$\mathcal{D}(\varphi,\varphi') \simeq \frac{1}{2\pi\nu\tau}\left[\frac{1}{\tau}\,\frac{1+\mathrm{i}ql(\cos\varphi+\cos\varphi') + (q^2l^2 - 2\mathrm{i}\omega\tau)\cos\varphi\cos\varphi'}{Dq^2 - \mathrm{i}\omega} + 2\sin\varphi\sin\varphi'\right], \tag{S33}$$

where ν is the density of states and the diffusion constant is given by $D = v_F^2\tau_\mathrm{tr}/2 = v_F^2\tau$ (note that in this model, for point-like impurities, the transport scattering time τ_tr is twice larger than the total scattering time τ).

The Cooperon ladder (that also starts with a single line) obeys the equation

$$\mathcal{C}(\varphi,\varphi') = W(\varphi-\varphi') + \frac{2\pi\nu\tau}{\hbar}\left\langle W(\varphi-\varphi_1)P(\varphi_1)\mathcal{C}(\varphi_1,\varphi')\right\rangle_{\varphi_1}. \tag{S34}$$

$$\tag{S35}$$

The resulting Cooperon propagator acquires an additional phase factor compared to the diffuson propagator (S33):

$$\mathcal{C}(\varphi,\varphi') = e^{i(\varphi'-\varphi)}\tilde{\mathcal{C}}(\varphi,\varphi') \tag{S36}$$

In the absence of dephasing,

$$\tilde{\mathcal{C}}(\varphi,\varphi') = \mathcal{D}(\varphi,\varphi'). \tag{S37}$$

We now use the propagators (S33) and (S36) to compute the interaction-induced corrections to the conductivity depicted in Fig. S1. The diagrams shown in Fig. S1 contain Hikami boxes, i.e., the insertions of the disorder line between two parallel retarded or advanced Green functions. The direction of the arrows of the Green functions gives rise to W and $\bar{W}$ factors. The phase difference of these factors matches the difference between Cooperon and diffuson ladders. As a result, the relative phase factors is completely cancelled. Therefore, in terms of $\tilde{C}$, each diagram is identical to those in Ref.[S8], but with an opposite sign that emerges from the π shift in the directions of the incoming and outgoing momenta. Therefore, the contribution of all diagrams in Fig. S1 for the dimensionless conductance of a single surface at $l_{\rm B} < l_T$ (equivalent to $B > \frac{k_B T}{De} = 4B_{\rm T}$) is given by[S5]

$$\delta g_1^{\rm CWAL} = -\frac{1}{\pi^2 g_1} \ln\left(\frac{l_{\rm B}}{l_{\rm tr}}\right). \tag{S38}$$

For $l_{\rm B} > l_T$ (equivalent to $B < \frac{k_B T}{De} = 4B_{\rm T}$) one gets[S8]:

$$\delta g_1^{\rm CWAL} = -\frac{3}{2\pi^2 g_1} \ln\left(\frac{l_{\rm B}}{l_{\rm tr}}\right). \tag{S39}$$

In the presence of external gate placed at the distance d from the sample, the lorarithm in Eqs. (S38) and (S39) is cut by the minimum of $l_{\rm B}$ and d.

2. Particle-particle (Cooper) channel

In addition to the interaction in the particle-hole channel discussed above, the interaction in the particle-particle channel (i.e., the Cooper channel) affects the conductivity. On the perturbative level, the dimensionless magneto-conductance $g_1(B,T)$ acquires corrections governed by the coupling constant γ_c that accounts for a Cooper ladder of original interactions lines. In the case of attractive interaction, this Cooper ladder describes superconducting fluctuations. For the repulsive interaction, it does not lead to superconducting instability, but nevertheless affects the conductance.

The full system of the RG equations that account for the Cooper channel was derived in Ref.[S6] (see also Ref.[S10]). In our analysis, we go up to the g^{-1} order, accounting for the effects of the second-loop WAL in Sections I A & I B and interaction-induced magnetoresistivity in the partcle-hole channel studied in Section I C 1. We introduce the parameter of the running scale $y = \log(\mathcal{L}/l_{\rm tr})$. In this approximation, the one-surface dimensionless conductance (for

$\mathcal{L} < l_{\rm B} < l_T$) for the long-range Coulomb repulsion is governed by the equations

$$\frac{dg_1}{dy} = \frac{1}{\pi} - \frac{1}{2\pi^2 g_1} + \frac{2\gamma_c}{\pi} \tag{S40}$$

$$\frac{d\gamma_c}{dy} = -2\gamma_c^2 + \frac{1}{\pi g_1}. \tag{S41}$$

The first term on the right hand side of Eq. S40 comes from non-interacting one-loop RG, in agreement with the HLN formula. The second term is a combination of two-loop non-interacting correction, Eq. (S21), and the cross-term accounting the interaction in the particle-hole channel, Eq.(S38). The last term is correction coming from particle-particle channel, which is commonly referred to as the DOS correction in the Cooper channel. The first term in Eq. (S41) accounts for the standard Cooper ladder of interactions (which describes the conventional superconducting instability at the mean-field level for attractive interaction, $\gamma_c < 0$). The second term in Eq. (S41) amounts for the renormalization of the coupling constant γ_c due to the interplay of disorder and Coulomb interaction[S8]. When the bare Cooper coupling is dominated by attraction, this term leads to the suppression of superconductivity in disordered systems.

Near the weak coupling limit ($g_1 \gg 1$ and $\gamma_c \ll 1$), one can solve equations (S40) and (S41) separately. With this accuracy, one finds

$$\gamma_c(y) = \frac{1}{\sqrt{2\pi g_1}} \tanh\left(\sqrt{\frac{2}{\pi g_1}} y + \varphi\right) \tag{S42}$$

Here, $\varphi = \text{arctanh}[\sqrt{2\pi g_1}\gamma_c(0)]$, and $\gamma_c(0) \sim \log^{-1}(T_c\tau) \ll 1$ is the value of the Cooper interaction constant at the ultraviolet limit $\mathcal{L} \sim l_{\rm tr}$, with $T_c \sim E_F \gg 1/\tau$. When the Fermi energy E_F is not too large, it is more convenient to express the initial condition for the RG flow through the Drude conductance: $\gamma_c(0) \sim \log^{-1}(g) \ll 1$.

In the weak-coupling limit, where both RG charges $1/g$ and γ_c are small, we infer from the Eq. S42 that γ_c remains small, as long as $\gamma_c(0)g_1 \gg \ln(l_{\rm B}/l_{\rm tr})$. One notes that in the infrared limit (for $y \to \infty$) this interaction constant flows to

$$\gamma_c \to 1/\sqrt{2\pi g_1} \tag{S43}$$

This is a universal value for the long-range interaction[S8]. Substituting Eq. (S42) into Eq. (S40), one finds the magnetoconductance of one surface (for $l_{\rm B} < l_T$)

$$g_1(B,T) = g_1^{(0)} + \left(\frac{1}{\pi} + \frac{1}{2\pi^2 g_1}\right) \ln\left(\frac{l_{\rm B}}{l_{\rm tr}}\right) - \frac{1}{\pi^2 g_1} \ln\left(\frac{l_{\rm B}}{l_{\rm tr}}\right) + \frac{1}{\pi} \ln\left(\frac{\cosh\left(\sqrt{\frac{2}{\pi g_1}} \ln\left(\frac{l_{\rm B}}{l_{\rm tr}}\right) + \varphi\right)}{\cosh\varphi}\right). \tag{S44}$$

In the weak coupling limit Eq. (S44) yields

$$g_1(B,T) = g_1^{(0)} + \left(\frac{1}{\pi} + \frac{1}{2\pi^2 g_1}\right) \ln\left(\frac{l_{\rm B}}{l_{\rm tr}}\right) - \frac{1}{\pi^2 g_1} \ln\left(\frac{l_{\rm B}}{l_{\rm tr}}\right) + \frac{2\gamma_c(0)}{\pi} \ln\left(\frac{l_{\rm B}}{l_{\rm tr}}\right) \tag{S45}$$

In this analysis, we keep track of logarithmic terms and ignore non-singular corrections, even if they depend on B. The dimensionless magnetoconductance of a single surface is thus given by

$$\Delta g_1(B) = \left(\frac{1}{\pi} + \frac{1}{2\pi^2 g_1}\right) \ln\left(\frac{l_{\rm B}}{l_\varphi}\right) + \left(\frac{2\gamma_c(0)}{\pi} - \frac{1}{\pi^2 g_1}\right) \ln\left(\frac{l_{\rm B}}{l_T}\right) \tag{S46}$$

for $l_{\rm B} < l_T < l_\varphi$.

Next we analyze the magnetoconductance in a weak magnetic field ($l_T < l_{\rm B}$). This implies a two-step RG procedure. The renormalization for the running scales $\mathcal{L} < l_T$ is followed by the renormalization with a modified beta-function at $l_T < \mathcal{L} < l_{\rm B}$. The first part of this procedure ($\mathcal{L} < l_T$) is described by Eqs. (S40) and (S41). At $l = l_T$ the flow of the interaction constant in the superconducting channel stops, determining the value of $\gamma_c = \gamma_c(y_T)$ for longer scales. Therefore, terms that are linear in γ_c no do not depend on $l_{\rm B}$, and do not contribute to magneto-conductance. In addition, the contribution of the interaction-induced magnetoconductance changes, see Eq. (S39). As a result, the second stage of the RG procedure ($l_T < \mathcal{L} < l_{\rm B}$) is performed with following RG equation:

$$\frac{dg_1}{dy} = \frac{1}{\pi} - \frac{1}{\pi^2 g_1}\,, \tag{S47}$$

resulting in

$$g_1(l_{\rm B}) = g_1(l_T) + \frac{1}{\pi} \ln\left(\frac{\min\{l_{\rm B}, l_\varphi\}}{l_T}\right) - \frac{1}{\pi^2 g_1} \ln\left(\frac{l_{\rm B}}{l_T}\right). \tag{S48}$$

The corresponding magnetoconductance for $l_T < l_{\rm B} < l_\varphi$ then reads:

$$\Delta g_1(B) = \left(\frac{1}{\pi} - \frac{1}{\pi^2 g_1}\right) \ln\left(\frac{l_{\rm B}}{l_T}\right). \tag{S49}$$

In addition, there are real transitions that are influenced by magnetic field and thus affect the MC. They are not accounted in the above RG procedure and should be added separately. These are the Maki-Thompson correction[S11,S12,S13] and dephasing processes. The Maki-Thompson corrections is quadratic in the amplitude γ_c and reads:

$$\delta g_1^{\rm MT} = -\frac{\pi}{3}\gamma_c^2 \ln\left(\frac{l_\varphi}{l_{\rm B}}\right). \tag{S50}$$

The dephasing time is affected by the magnetic field, see Eq. S54 below. This influences the HLN term in Eq. S44 and thus the MC.

3. Dependence of the dephasing time on magnetic field

The standard HLN term in Eq. S44,

$$\delta g_1^{\rm HLN} = \frac{1}{\pi}\ln\left(\frac{\min\{l_{\rm B}, l_\varphi\}}{l_{\rm tr}}\right), \tag{S51}$$

was computed so far with a logarithmic accuracy. It is known that a more careful computation yields

$$\delta g_1^{\rm HLN} \to \frac{1}{2\pi}\left[\Psi\left(\frac{1}{2}+\frac{l_{\rm B}^2}{l_{\rm tr}^2}\right) - \Psi\left(\frac{1}{2}+\frac{l_{\rm B}^2}{l_\varphi^2}\right)\right] \simeq \frac{1}{2\pi}\left[\ln\left(\frac{1}{\Omega_B\tau}\right) - \frac{1}{\Omega_B\tau_\varphi}\right], \quad \text{for} \quad l_{\rm tr} \ll l_{\rm B} \ll l_\varphi. \tag{S52}$$

Here, $\Omega_B = 4DeB/\hbar$. The dephasing rate in the presence of the magnetic field is given by

$$\frac{1}{\tau_\varphi(B)} = \frac{T}{g}\log\left(\frac{T}{\max\{\Omega_B, 1/\tau_\varphi\}}\right), \tag{S53}$$

which induces an additional dependence of the HLN term on B in the range $T \gg \Omega_B \gg 1/\tau_\varphi$:

$$\delta g_1^{\rm HLN} \simeq \frac{1}{2\pi}\left[\log\frac{1}{\Omega_B\tau} - \frac{\pi^2 T}{2g\Omega_B}\log\frac{T}{\Omega_B}\right] \tag{S54}$$

The fist term corresponds to the standard one-loop HLN formula, while the second term generates an additional B-dependent contribution to the conductance. Though it is typically small, it is proportional to $1/g$ and, therefore, needs to be included in our analysis. This correction should be added to the MC in the regime $l_{\rm B} > l_T$.

D. Summary of theoretical results

We now summarize the final formulas derived above for the total two-surface MC. In the absence of external gates, the MC reads

$$\Delta g^{\rm total}(B) = 2\left(\frac{1}{\pi} - \frac{\pi}{2g}\frac{l_{\rm B}^2}{l_T^2} + \frac{\pi}{3}\gamma_c^2 - \frac{1}{\pi^2 g_1}\right)\log\left(\frac{l_{\rm B}}{l_T}\right), \quad \text{for} \quad l_\varphi > l_{\rm B} > l_T > l_{\rm tr}\,, \tag{S55}$$

$$\Delta g^{\rm total}(B) = 2\left(\frac{1}{\pi} + \frac{1}{2\pi^2 g_1}\right)\ln\left(\frac{l_{\rm B}}{l_\varphi}\right) + 2\left(\frac{2\gamma_c(0)}{\pi} - \frac{1}{\pi^2 g_1}\right)\ln\left(\frac{l_{\rm B}}{l_T}\right), \quad \text{for} \quad l_\varphi > l_T > l_{\rm B} > l_{\rm tr}\,. \tag{S56}$$

In the presence of the gate, placed at distance d from the sample, the CWAL term does not produce a logarithmic dependence on magnetic field for $l_{\rm B} > d$. In this case, the equations above are modified as follows:

$$\Delta g^{\rm total}(B) = 2\left[\frac{1}{\pi} - \frac{\pi}{2g}\frac{l_{\rm B}^2}{l_T^2} + \frac{\pi}{3}\gamma_c^2 + \frac{1}{2\pi^2 g_1}\right]\log\left(\frac{l_{\rm B}}{l_T}\right), \quad \text{for} \quad l_\varphi > l_{\rm B} > l_T > l_{\rm tr}\,, \tag{S57}$$

$$\Delta g^{\rm total}(B) = 2\left(\frac{1}{\pi} + \frac{1}{2\pi^2 g_1}\right)\ln\left(\frac{l_{\rm B}}{l_\varphi}\right) + \frac{4\gamma_c(0)}{\pi}\ln\left(\frac{l_{\rm B}}{l_T}\right), \quad \text{for} \quad l_\varphi > l_T > l_{\rm B} > l_{\rm tr}\,. \tag{S58}$$

Equations (S57) and (S58) correspond to the situation realized in the experiment and are used to fit the data in the main part of the manuscript, assuming interaction constant is close to the universal limit,

$$\gamma_c \simeq \frac{1}{\sqrt{\pi g}}.$$

This is justified if the bare value at ultraviolet limit $\gamma_c(0)$ is large enough, such that the RG flow comes close to the universal limit at scale of the order d.

II. SUPPLEMENTARY NOTE 2

Evaluation of the effective aspect ratio with finite element analysis

As shown in Fig. S2, the device geometry used in this work is not the standard Hall bars. The irregular sample shapes, as well as the overlap between metal electrodes and TI flakes, preclude a straightforward conversion of the four-point resistance $R_{\rm xx}$ to the sheet longitudinal conductivity $\sigma_{\rm xx}$. We apply the finite-element analysis to simulate the electric potential distribution[S14], which serves as a basis for reliable evaluation of $\sigma_{\rm xx}$.

The task of the numerical simulation is to find the geometrical factor f_s (i.e., number of equivalent squares for the quasi-2D transport) for each four-point resistance measurement. For instance, when a current is applied between the source and drain (denoted as electrodes 5 and 6 in Fig. S3), the voltage measured between electrodes 1 and 2 determines the four-point resistance $R_{56,12}$. The resistance per square follows

$$R_{\square} = \frac{1}{\sigma_{\rm xx}} = \frac{1}{f_s} R_{\rm xx} = \frac{1}{f_s} \frac{V_{12}}{I_{56}}. \tag{S59}$$

In our devices the electrical contacts are only in touch with the bottom surface of the TI flakes, so there might be considerable asymmetry in the current flow and the electrical field distribution between the top and bottom surfaces. The numerical analysis is further complicated by the irregular sample shape, ratio between the surface and bulk conductivities, as well as contact resistances and their local variations. Nevertheless, the simulation can be simplified by working out two extreme cases. One is the low bulk-conductivity limit, at which the bulk resistivity is assigned with a value exceeding the upper bound and the surface resistivity takes the minimum value observed in the experiment. The other is the high bulk-conductivity limit, at which the bulk and surface resistivities are set at their lower and upper bounds, respectively. When the transport parameters are varied from the first limit (surface-dominating) toward the second one (bulk-dominating), the asymmetry between the top and bottom surfaces is expected to decrease. As will be shown below, these two limits exert a strong constraint on the uncertainty level of evaluating the geometrical factors.

Fig. S3(a) depicts the electric potential distribution on the top and bottom surfaces of sample A at the limit of low bulk conductivity and high surface conductivity. The bulk resistivity is estimated to be 1-10 Ω·cm for our BSTS samples, so we use $\rho_{\rm xx} = 20$ Ω·cm in the finite element analysis in order to be on the safe side. The sheet resistivity of sample A is about 2-8 kΩ (or 4-16 kΩ each surface). The TI surface is simulated with a 1 nm thick layer with high 3D conductivity, but the sheet resistance is fixed at 4 kΩ per square for each surface. In contrast, the sheet resistivity

of the bulk layer at this limit would be 20 Ω·cm/26 nm≈ 7.7 MΩ, three orders magnitude larger than the surface resistivity (∼2 kΩ, when the contributions from the top and bottom surfaces are included).

Shown in Fig. S3(b) is the electric potential distribution at the limit of high bulk conductivity and low surface conductivity. A bulk resistivity of 1 Ω·cm and a surface resistivity of 8 kΩ are used in the simulation. The overall electric field potential distribution, however, varies very little in comparison with that in the previous regime (i.e. low bulk/high surface conductivity, see Fig. S3(a)).

Here, we introduce a parameter γ to characterize the asymmetry between the top and bottom surfaces. It is defined as

$$\gamma_i = \left| \frac{V_{i,\mathrm{t}} - V_{i,\mathrm{b}}}{V_{1,\mathrm{b}} - V_{2,\mathrm{b}}} \right| , \tag{S60}$$

where $V_{i,\mathrm{t}}$ and $V_{i,\mathrm{b}}$ denote the electric potential on electrode i on the top and bottom surfaces, respectively. For sample A, γ is in a range of 0.02% to 0.18% at the low bulk conductivity limit, whereas γ is less than 0.01% for each of the electrode at the high bulk conductivity limit. The small γ values can be attributed to the quasi-2D nature of sample A, in which the TI flake is only 26 nm thick, nearly three orders of magnitude smaller than its lateral sizes. Moreover, the bulk of BSTS material is not a perfect insulator, and this helps reduce the asymmetry between the two surfaces. As shown in Fig. S3c&d, the current distribution in the central area is almost uniform, only slightly influenced by the electrical contacts. We therefore conclude that the asymmetry effect can be largely neglected during the evaluation of the geometrical factor for longitudinal transport. The finite element analysis yields f_s=0.61-0.63, very close to $f_s = 0.60$ obtained by the straightforward geometrical consideration, in which the voltage probes are treated as ideal contacts, and thus do not modify the current flow.

When the magnetic field is applied, the above analysis will remain valid if the Hall angle, defined as $\theta_\mathrm{H} = \rho_\mathrm{xy}/\rho_\mathrm{xx} = \mu B$, is much smaller than 1, where μ is the carrier mobility. As depicted in Fig. S4d, θ_H is less than 0.07 at $B = 0.5$ T for all gate voltages. Beyond 0.5 T, the weak antilocalization effect is suppressed significantly by the magnetic field. Since in this work the HLN fits are mostly restricted to the fields below 0.25 T, it is not necessary to consider the complications caused by the large Hall angles.

In principle, one could also use the finite element analysis to obtain the geometric factor for the Hall resistivity, defined as $f_\mathrm{H} = 1/(n_s q R_\mathrm{H})$. Here, n_s is the sheet carrier density, q is the carrier's charge, and R_H is the Hall coefficient. The Hall voltages are, however, prone to the influences of metal electrodes, as they extend considerably into the interior of TI flakes. The uncertainty level of f_H is expected to be much higher than its longitudinal counterpart. Fortunately, the carrier density can be extracted by utilizing the electrostatic field effect (i.e., the capacitive coupling), with the

gating efficiency calibrated with quantum oscillations. As shown in Table I, f_{H} varies significantly, from 3.25 in sample A to 2.02 in sample C. Due to the above complications, we do not use the Hall effect data to extract the carrier densities in this work.

III. SUPPLEMENTARY NOTE 3

Summary of basic parameters of samples A, B and C

TABLE I: Basic transport parameters of three BSTS devices (samples A, B and C).

Sample No.	TI thickness (nm)	BN thickness (nm)	ρ_{xx} max Ω	V_{BG} & V_{TG} for ρ_{xx} max (V)	Raw R_H max Ω/T	Raw R_H min Ω/T	f_s (FEA)	f_s (geometry)	f_H
A	26	54	8300	-21 & 0.6	890	-710	0.60	0.61	3.25
B	64	40	6488	2 & -0.9	575	-535	0.77	0.76	2.48
C	46	52	6579	-50 & -4.9	390	-364	0.58	0.58	2.02

Table I displays some basic transport properties of three BSTS devices, from which the data in the main text was collected. The minimum thickness of the BSTS layer is 26 nm, so that the hybridization effect between the top and the bottom surfaces can be neglected. The BN layer in each device is 40-54 nm thick. For sample A, the distances from the metallic top-gate to the top and bottom surfaces are $d_1 = 54\,\text{nm}$ and $d_2 = 80\,\text{nm}$, respectively. They correspond to magnetic fields $B_{d,1} = \sqrt{\hbar/(ed_1^2)} \approx 0.23\,\text{T}$ and $B_{d,2} = \sqrt{\hbar/(ed_2^2)}] \approx 0.10\,\text{T}$, below which the screening effect of the top gate starts to suppress the MC correction due to the cross-term in the particle-hole channel (see Supplementary Note 1, section I D). Similar effects are also expected for samples B and C.

IV. SUPPLEMENTARY NOTE 4

Further characterization of sample A

Fig. S4 shows more basic transport data complementary to Fig. 1. Eight gate-voltage sets (points 1-8, along the symmetric gating line depicted in Fig. S4**a**) are selected to show the Hall resistance curves for magnetic fields up to 2 T (Fig. S4**b**)). The R_{xy} curves exhibit good linearity for $V_{\mathrm{BG}} < -30$ V or $V_{\mathrm{BG}} > -15$ V. For the gate voltages in between, the Hall resistance deviates from the linear dependence. This can be ascribed to the formation of electron and hole puddles due to the long-range disorder. This type of transport is often referred to as the ambipolar regime in the literature.

Fig. S4**c** depicts the gate-voltage dependence of the nominal carrier density extracted from the raw Hall coefficient, defined as $n_{\mathrm{s,H}} = 1/(-eR_{\mathrm{H}})$, where R_{H} is evaluated with the R_{yx} value at $B = 1\,\mathrm{T}$, and the minus sign in the denominator gives positive $n_{\mathrm{s,H}}$ values for electrons. It shows that $n_{\mathrm{s,H}}$ exhibits a nearly linear dependence for the gate voltages well separated from the charge neutral point ($V_{\mathrm{BG}} < -30$ V or $V_{\mathrm{BG}} > -15$ V), indicating the transport dominated by either holes or electrons, labeled as zone I and zone III. It should be noted, however, $n_{\mathrm{s,H}}$ differs from the true carrier density by a geometrical factor (f_{H}, see Table I). The strong deviation from linear dependence in zone II also originates from the ambipolar nature of the transport, in which the $n_{\mathrm{s,H}}$ value would be significantly different from the real carrier density, even if the uncertainty in f_{H} could be ignored. Shown in Fig. S4**d** are the Hall angles at $B = 0.5$ T for a set of gate voltages along the symmetric gating line. At $V_{\mathrm{BG}} = -15$ V, θ_{H} reaches a maximum value of 0.07.

TABLE II: Transport parameters of sample A.

V_G-Point	V_{BG} (V)	R_{sq} (Ω)	$g^{(0)}$ ($\equiv k_F l_{\mathrm{tr}}$)	n_s ($10^{12}\mathrm{cm}^{-2}$)	k_F (nominal) (nm^{-1})	k_F (effective) (nm^{-1})	ν_F (10^5 m/s)	l_{tr} (nm)	τ_{tr} (fs)	D ($10^{-3}\mathrm{m}^2/\mathrm{s}$)
A	-25.0	7326	3.52	-0.291	0.135	0.122	4.83	26.1	54.0	6.30
B	-20.0	7848	3.29	0.019	0.035	0.121	4.98	27.2	54.6	6.77
C	-17.5	7138	3.62	0.174	0.105	0.132	5.05	27.4	54.3	6.92
D	-7.0	3868	6.67	0.824	0.227	0.227	5.16	29.4	57.0	7.59
E	31.0	1815	14.2	3.12	0.443	0.443	5.35	32.1	60.0	8.59

For a 2D Dirac fermion system, the short-range scattering would lead to transport lifetime $\tau_{\rm tr}$ satisfying

$$\frac{1}{\tau_{\rm tr}} = n_{\rm imp}\frac{k_F}{\pi\hbar^2 v_F}\int_0^\pi |\tilde{V}_0|^2(1-\cos\theta)\frac{1+\cos\theta}{2}d\theta \propto k_F \propto n^{1/2}, \tag{S61}$$

which leads to $R_{\rm xx}$ independent of k_F or carrier density. In contrast, the Coulomb interaction in 2D results in

$$\frac{1}{\tau_{\rm tr}} \propto n_{\rm imp}\frac{1}{k_F} \propto n^{-1/2}, \tag{S62}$$

which is valid under the Thomas-Fermi approximation. In the latter case, one would expect $R_{\rm xx} \propto k_F^{-2} \propto n^{-1}$. As depicted in Fig. S5, $R_{\rm xx}$ is approximately proportional to $n^{-0.57}$ if the electron density is not too low (i.e., outside of the ambipolar region). The momentum relaxation time is thus nearly independent of the carrier density, consistent with the $\tau_{\rm tr}$ values displayed in Table II. On one hand, such a carrier density dependence is different from graphene, which exhibits the typical behavior of 2D Coulomb potential[S15]. On the other hand, it also differs greatly from the isotropic short-range scattering. This unique behavior is probably related to the long-range electric potential fluctuations induced by the compensation doping in the BSTS, which has been considered as a driving force for the formation of electron and hole puddles in the bulk.

V. SUPPLEMENTARY NOTE 5

Multiple length scales and analysis of quantum corrections to MC

A. Summary of MC corrections of various length scales

As discussed in Supplementary Note 1, the MC of TI surface states can be modified by several types of quantum corrections, including the first- and second-order interference effects, Maki-Thompson (MT) and the density of states (DOS) interaction corrections in the particle-particle channel, and the mutual effect of the WAL and electron-electron interaction (EEI) in the particle-hole channel (i.e., the cross-term correction, denoted as CWAL, which has a counterpart in an electron system with the orthogonal symmetry[S5]). In general, it is quite challenging to quantitatively account for the MCs in TIs because these corrections are related to multiple length scales (see Supplementary Note I and Table III). The quantum interference effects are determined by the competition between dephasing length $l_\varphi = \sqrt{D\tau\varphi}$ and magnetic length l_B, when $l_B = \sqrt{2\pi\hbar/(eB)}$ is much longer than the mean free path l_tr. The MT correction depends on both l_φ/l_B and l_T/l_B, where $l_T = \sqrt{\hbar D/(k_B T)}$ is the thermal length. The DOS correction is much smaller than the MT term in weak magnetic fields. When $l_B > l_T$, it crosses over to a $\ln(B/B_\mathrm{T})$ dependence, with the characteristic field defined as $B_\mathrm{T} = \hbar/(4el_T^2)$. The cross-term correction leads to positive MCs for TI surface states, opposite to all other quantum corrections mentioned above. For $l_B \ll l_\varphi$, $\Delta\sigma_\mathrm{CWAL}$ follows a $\ln(B/B_\mathrm{tr})$ dependence, but with a coefficient varies from $3/(2\pi g)$ to $1/(\pi g)$ as the magnetic field increases from a regime of $l_B > l_T$ to $l_B < l_T$ (see Ref.[S5] and Section I C 1). When a metallic gate is nearby, the cross-term correction is susceptible to the screening effect and starts to saturate at $l_\mathrm{B} \sim d$ (in other words, $\Delta\sigma_\mathrm{CWAL}$ gets suppressed for $B < \sqrt{2\pi\hbar/(ed^2)}$). In this work, d refers to the distance between a TI surface and the metallic top-gate (a Cr-Au layer), since the bottom gate is a silicon substrate separated from the TI flake by a 300 nm thick SiO_2 layer.

Since the analysis of conductance corrections is often carried out with respect to the magnetic field, it is more convenient to discuss the relevant physics in a scale of magnetic field. Table III gives the definition of the characteristic B-fields corresponding to the aforementioned length scales, and when the magnetic field is sufficiently strong, so that $l_B < l_\varphi$ and $l_B < l_\mathrm{T}$ are satisfied, and at the same time B is weak enough to stay diffusive (i.e., $l_B > l_\mathrm{tr}$), each of the MC corrections approaches a $\ln B$-type dependence asymptotically (see Supplementary Note 1, SectionI D, and Ref. [S5]). Following the definitions of these length scales in Section I (summarized in Table III), the crossover conditions

TABLE III: Various characteristic lengths, magnetic fields and times related to the quantum corrections to MC. Please note that the definition of l_B is different from most of the literature by a factor of 2π by following the convention adopted in Section I A.

Characteristic length	Characteristic magnetic field	Characteristic time	Relation
dephasing length l_φ	dephasing field B_φ	dephasing time τ_φ	$B_\varphi = \frac{\hbar}{4el_\varphi^2} = \frac{\hbar}{4De\tau_\varphi}$
mean free path $l_{\rm tr}$	transport field $B_{\rm tr}$	momentum relaxation time $\tau_{\rm tr}$	$B_{\rm tr} = \frac{\hbar}{2el_{\rm tr}^2} = \frac{\hbar}{4De\tau_{\rm tr}}$
thermal length $l_{\rm T} = \sqrt{De/(k_BT)}$	thermal field $B_{\rm T}$	thermal scattering time $\tau_{\rm T} = \hbar/(k_BT)$	$B_{\rm T} = \frac{\hbar}{4el_{\rm T}^2} = \frac{k_BT}{4De}$
magnetic length $l_{\rm B}$	magnetic field B	magnetic time $\tau_{\rm B} = 1/\Omega_B$	$l_{\rm B} = \sqrt{\hbar/(eB)}$ $\Omega_B = 4DeB/\hbar$

$l_B \sim l_\varphi$ and $l_B \sim l_{\rm T}$ are equivalent to $B \sim \frac{\hbar}{De\tau_\varphi} = 4B_\varphi \approx 2\pi B_\varphi$ and $B \sim \frac{k_BT}{De} = 4B_{\rm T} \approx 2\pi B_{\rm T}$, respectively.

B. Analysis of the MC corrections

Although the experimental MC often exhibits the ln$B$-like behavior in sufficiently high magnetic fields, it is very difficult to identify the sources of quantum corrections by carrying out a logarithmic fit. This can be attributed to the following two reasons: 1) the magnetic field range is too narrow or non-existent to perform a meaningful fit, if both $B \gg 2\pi B_{\rm T}$ (equivalent to $l_{\rm B} \ll l_{\rm T}$) and $B \ll B_{\rm tr}$ are satisfied with rigor, as shown in Table IV; 2) the MC curves at $B \gg B_{\rm T}$ usually don't contain sufficient curvatures to allow for a reliable extraction of over two fitting parameters, and hence insufficient to differentiate so many different sources of conductance corrections.

In order to disentangle various types of MC corrections, we fit the MC data in low magnetic fields to Eq. (1), typically in a range of B = 0-15B_φ. This field range satisfies $B \ll B_{\rm tr}$ for the TI samples studied in this work (see Table IV). Another benefit of this fitting range is that the MC curves possess adequate curvatures to obtain the fitting parameters (α and B_φ) reliably. More importantly, the MC curves follow or only slightly deviates from the Eq. (1) in such low magnetic fields. Given the fact that MC corrections from the first- and second-order quantum interference effects are well described with Eq. (1) (see below for further justification), the total contribution of various EEI effects

can be approximately casted into a form of the $Y(B/B_\varphi)$-function (defined as $Y(x) = \psi(1/2 + 1/x) + \ln x)$), at least for $B < 15B_\varphi$. Since none of the MT, DOS and cross-term corrections take a form of $Y(B/B_\varphi)$-function rigorously, the good agreement between the experimental MC curves and the HLN fits (simplified for low fields) is worth some discussion.

TABLE IV: Characteristic magnetic fields and length scales relevant to the quantum corrections to the MC in sample A at $T = 1.6\,\mathrm{K}$.

Gate-voltage point	V_{BG} (V)	g ($\equiv k_F l$)	l (nm)	l_φ (nm)	l_T (nm)	B_φ (mT)	$2\pi B_{\mathrm{T}}$ (mT)	$B_{\max}(= 15B_\varphi)$ (mT)	B_{tr} (mT)	α	D ($10^{-3}\mathrm{m}^2/\mathrm{s}$)
A	-25.0	3.52	26.1	223	173	3.31	34.5	49.7	482	1.02	6.30
B	-20.0	3.29	27.2	257	179	2.50	32.0	37.5	444	1.29	6.77
C	-17.5	3.62	27.4	286	181	2.01	31.4	30.2	437	1.40	6.92
D	-7.0	6.67	29.4	370	190	1.20	28.3	18.0	380	1.26	7.59
E	31.0	14.2	32.1	462	202	0.77	25.1	11.6	318	1.08	8.59

As the mentioned in main text, the MT correction is proportional to the $Y(B/B_\varphi)$ function when $B \ll 2\pi B_{\mathrm{T}}$ (see (Eq. (2)) in the main text). For a wider range of magnetic fields, the MT term can be written as[S5]

$$\frac{\Delta\sigma_{\mathrm{MT}}}{G_0} = \pi\Delta g_{\mathrm{MT}} \simeq -\frac{\pi^2}{6}\gamma_c \left[Y(\frac{B}{B_\varphi}) - Y(\frac{B}{2\pi B_{\mathrm{T}}}) \right], \tag{S63}$$

with $G_0 = e^2/(2\pi^2\hbar)$. This equation is similar to the MC correction due to the quantum interference effects:

$$\frac{\Delta\sigma_{\mathrm{QI}}}{G_0} = -\alpha_{\mathrm{QI}} \left[Y(\frac{B}{B_\varphi}) - Y(\frac{B}{B_{\mathrm{tr}}}) \right], \tag{S64}$$

in which B_{tr} replaces $2\pi B_{\mathrm{T}}$ in Eq. S63, and the prefactor satisfies $\alpha_{\mathrm{QI}} \simeq (1 + 1/(\pi g))$ for the TI surface states, with 1 and $1/\pi g$ in the bracket originating from the first- and second-order quantum interferences, respectively. In Eqs. S63 and S64, the second term leads to saturation of the MC at high magnetic fields ($B \gg 2\pi B_{\mathrm{T}}$ or $B \gg B_{\mathrm{tr}}$). In weak magnetic fields ($B \ll 2\pi B_{\mathrm{T}}$ or $B \ll B_{\mathrm{tr}}$), these two terms are negligible and these two equations reduce to Eqs. (2) and (1) in the main text.

As shown in Table IV, the condition $B \ll B_{\mathrm{tr}}$ can be satisfied for the field range B = 0-15B_φ, so the fits to Eq. (1) are justified for the conductance corrections caused by quantum interference. In contrast, the $2\pi B_{\mathrm{T}}$ value is comparable to the maximum magnetic field ($B_{\max} = 15B_\varphi$) for a typical fit. As a result, the B_{T} term cannot be ignored and the magnitudes of MC are expected to be slightly smaller than those given by Eq. (2). As depicted

in Fig. S6, the $B_{\rm T}$ term leads to nearly quadratic corrections to the MC in low magnetic fields. Its influence on the HLN fit is therefore quite weak for the fitting range 0-15B_φ, but expected to be more substantial as the fitting range is expanded. This effect is indeed borne out in our experimental observation. As shown in Fig. 3c, the α values of electron side decrease slightly as the fitting range increases from 0-15B_φ to 0-25B_φ. Similar results are also obtained for fitting ranges 0-10B_φ and 0-15B_φ (Fig. S7). Despite the small variations in the α value for different fitting ranges, the overall gate-voltage dependences of α have some common characteristics, such as a pronounced maximum appearing near the charge neutral point, enhancement of α diminishing at large electron densities, and α values nearly independent of the fitting range on the hole side.

As pointed out in Ref.[S5], at higher magnetic fields ($B \gg 2\pi B_{\rm T}$, or equivalently, $\Omega_B \gg 2\pi T$), the MT correction vanishes in an asymptotic form different from Eq. S63. However, this subtle difference is negligible, since the MT correction is overwhelmed by the DOS term under this circumstance. The DOS correction is quadratic and overwhelmed by the MT term in low magnetic fields. For $B \gg 2\pi B_{\rm T}$, the DOS correction approaches

$$\Delta\sigma_{\rm DOS} \simeq \gamma_c(0)\ln\left(\frac{B}{2\pi B_{\rm T}}\right). \tag{S65}$$

Since the MC correction due to the MT and DOS corrections have the same sign, the decreasing MT contribution at large B is partially compensated by the DOS term, making the total Cooper-channel correction only slightly deviate from the single $Y(B/B_\varphi)$ function (i.e., Eq. (2)). Nevertheless, we also performed the HLN fits of the DOS correction alone with different fitting ranges. It was found that increasing the field range leads to an increase in the α value, instead of the decreasing α observed in the experiment. We therefore conclude that the contribution of DOS term is marginal for the fitting range 0-15B_φ.

As to the cross-term in the particle-hole channel, its correction to MC, $\Delta\sigma_{\rm CWAL}$, is suppressed by the screening effect of the metallic top-gate. As shown in Table I, in sample A the top metallic gate is separated from the top and bottom surfaces by distances of $d_1 = 54\,$nm and $d_2 = 80\,$nm, respectively. They correspond to magnetic fields of about 0.23 T and 0.10 T, covering most of the field ranges of the HLN fits carried out in this work.

It is also worthy noting that the B_φ and $B_{\rm T}$ values in Table IV are given for the data taken at $T = 1.6\,$K. Nevertheless, the above statement remains valid for other temperatures, because both B_φ and $B_{\rm T}$ increase linearly with T and their ratio remains nearly constant. If the conductance g is not too small, the dephasing rate can be approximately written as

$$\frac{1}{\tau_\varphi} \simeq \frac{k_B T}{\hbar}\frac{\ln g}{g}. \tag{S66}$$

It follows that

$$\frac{B_\varphi}{B_\mathrm{T}} \simeq \frac{\ln g}{g}, \tag{S67}$$

which is independent of the temperature.

In this work, the g values are in a range of about 3-14, corresponding to $2\pi B_\mathrm{T}$ values roughly 10-33 times B_φ (or $4B_\mathrm{T} \approx$ 7-21B_φ). The degree of overlap between $B > 4B_\mathrm{T}$ (equivalent to $l_\mathrm{B} < l_\mathrm{T}$) and the HLN fitting range (typically $B < B_\mathrm{max} = 15B_\varphi$) is a function of $\ln g/g$ and decreases with increasing g. The DOS correction is thus expected to vanish at the large g limit. On the other hand, the MC corrections caused by various EEI effects also diminish at $g \gg 1$. As shown in Eq. S57, both the second-order interference and MT corrections, have a prefactor scaled with $1/g$.The cross-term correction has a prefactor proportional to $1/g$ as well (see Eq. S39). Even if the screening by the metallic top-gate could not fully suppress the CWAL term, a $1/g$-type dependence would also appear, similar to the case of orthogonal symmetry[S5]. In summary, all of these corrections to the MC decrease monotonically with $1/g$, providing a direct explanation of the nearly linear dependence of α on $1/g$ depicted in Fig. 4.

C. Irrelevance of the dephasing term

Finally, we remark on possible MC corrections of the dephasing term (i.e., the second term in Eq. S55). Although the magnetic field dependence of dephasing rate may contribute to the MC (Supplementary Note 1, Section I C 3), this correction requires that $B \ll 2\pi B_\mathrm{T}$ and $B \gg B_\varphi$ to be satisfied simultaneously. As shown in Table IV, this condition cannot be met due to the modest g values encountered in this work. Therefore, this dephasing term can be dropped out in our discussion.

VI. SUPPLEMENTARY NOTE 6

Negligible influence of quadratic magnetoresistance background on the HLN fits

The quadratic and linear magnetoresistances (MRs) have also been observed in topological insulators previously by many groups[S16,S17,S18]. Among 3D topological insulators, the most pronounced linear MR was observed, in a very wide range of magnetic fields, in Bi_2Te_3[S16,S18]. However, it was also shown that the linear MR crosses over to a parabolic dependence at sufficiently low fields (typically of the order 0.1 T), when the WAL effect is suppressed by raising the temperature to about 10 K[S16]. For BSTS, no clear evidence has been reported so far for this type of linear MR, despite extensive studies (see, for instance, Refs.[S19,S20] and Fig. S8). The quadratic MR is thus expected to exist in a wider range of magnetic fields in BSTS than Bi_2Te_3. In this work, we are mainly concerned with the MC corrections related to the WAL effect. The corresponding data analysis is limited to magnetic fields lower than 0.25 T. The low-field quadratic background can be obtained by fitting the MR data taken at high magnetic fields (4-9 T for sample A, see Fig. S8). After this background is subtracted, the low-field MR data is converted to the MCs and fitted to the HLN equation (or its generalization). Table V shows that the result of HLN fit is barely modified by the removal of quadratic background.

TABLE V: Comparison of the fitting parameters extracted from the HLN fits with (α and B_φ) and without (α' and B'_φ) the high-field MR background. A self-consistent field range of $B = 0 - 15B_\varphi$ was used for the fits.

Gate points	α	B_φ (G)	α'	B'_φ (G)
A	1.023	34.24	1.022	34.21
B	1.286	27.32	1.285	27.30
C	1.403	23.31	1.403	23.32
D	1.264	14.13	1.262	14.11
E	1.082	8.148	1.082	8.162

VII. SUPPLEMENTARY NOTE 7

Influence of asymmetry between the top and bottom surfaces on the HLN fits

If the top and bottom surfaces are not equivalent, the analysis of MCs would be further complicated by such an asymmetry. Although a lot of efforts have been used to prevent the TI surface states from degradation during the device fabrication process, it is impossible to ensure that these two surfaces are perfectly identical in all aspects. At the temperatures of interest to this work, EEI is the main source of electron dephasing, and the dephasing time τ_φ can be determined from the conductivity. The corresponding dephasing field is given by

$$B_\varphi = \frac{\hbar}{4el_\varphi^2} = \frac{\hbar}{4De\tau_\varphi} = \frac{\hbar}{2ev_F^2\tau\tau_\varphi}, \tag{S68}$$

where l_φ is the dephasing length, and the diffusion coefficient is given by $D = \frac{1}{2}\nu_F^2\tau$. If the bulk does not contribute, low field MC at $g \gg 1$ limit can be written as

$$\Delta\sigma(B) \simeq \sum_i -\frac{1}{2}\frac{e^2}{2\pi^2\hbar}\left[\psi\left(\frac{1}{2}+\frac{B_{\varphi,i}}{B}\right) - \ln\left(\frac{B_{\varphi,i}}{B}\right)\right], \tag{S69}$$

where i =1,2 is the surface index. If the top and bottom surfaces are identical, one expects $B_{\varphi,1} = B_{\varphi,2}$, the above expression will reduce to Eq. (1) in the main text with α =1. When the two surfaces are not equivalent, the MC in principle cannot be described perfectly with Eq. (1). Nevertheless, it has been found by many studies that low field MC in many TI samples can be fitted very well with Eq. (1) by treating both α and are B_φ as free parameters. The α values obtained from such fits are mostly smaller than 1. Fig. S9 shows that α decrease monotonically as the ratio $B_{\varphi,1}/B_{\varphi,2}$ increases. When the asymmetry between the two surfaces is not too large, the correction to α is quite modest. For instance, α becomes 0.963 and 0.9045 for $B_{\varphi,1}/B_{\varphi,2}$=2 and 3, respectively. Moreover, the α value for the asymmetric case ($B_{\varphi,1}/B_{\varphi,2} \neq 1$) is always smaller than that for the symmetric case ($B_{\varphi,1}/B_{\varphi,2} = 1$). This statement remains valid when the second loop and MT corrections are included because they are described with the function of same length scales (l_B and l_φ).

VIII. SUPPLEMENTARY NOTE 8

Evaluation of the Fermi velocity with the dephasing rate

Dephasing field B_φ is the other important parameter extracted from the HLN fits. Figure 2d shows the temperature dependence of B_φ for the five representative gate-voltage points. Each of them can be fitted to a linear function $B_\varphi = b_\varphi T + B_0$. It is generally accepted at temperatures below 10 K, EEI is the dominant source of dephasing, and the dephasing time τ_φ in a 2D system follows

$$\frac{k_B T \tau_\varphi}{\hbar} \ln\left(\frac{k_B T \tau_\varphi}{\hbar}\right) = g. \tag{S70}$$

This leads to the linear temperature dependence of dephasing rate $1/\tau_\varphi$, which is presumably responsible for the T-dependent part of B_φ, namely $B_\varphi^* \equiv b_\varphi T = \hbar/\left(4De\tau_\varphi^{\mathrm{EEI}}\right)$. Fig. S10 depicts the dephasing rates calculated self-consistently with Eq. S70 for the five gate-voltage points. Assuming b_φ, the slope of each B_φ-T curve in Fig. 2d, being solely contributed by the EEI-induced dephasing, we obtain

$$\left(\tau_\varphi^{\mathrm{EEI}}\right)^{-1} = \frac{2ev_F g b_\varphi T}{\hbar k_F}. \tag{S71}$$

All parameters on the right-hand side of the above equation can be determined by the transport measurements except v_F. Using theoretical values of $\tau_\varphi^{\mathrm{EEI}}$, we could deduce v_F with

$$v_F = \frac{\hbar k_F \left(\tau_\varphi^{\mathrm{EEI}}\right)^{-1}}{2egb_\varphi T}. \tag{S72}$$

The Fermi velocities of points D and E are evaluated to be 4.2×10^5 m/s and 4.0×10^5 m/s, respectively.

IX. SUPPLEMENTARY FIGURE S1

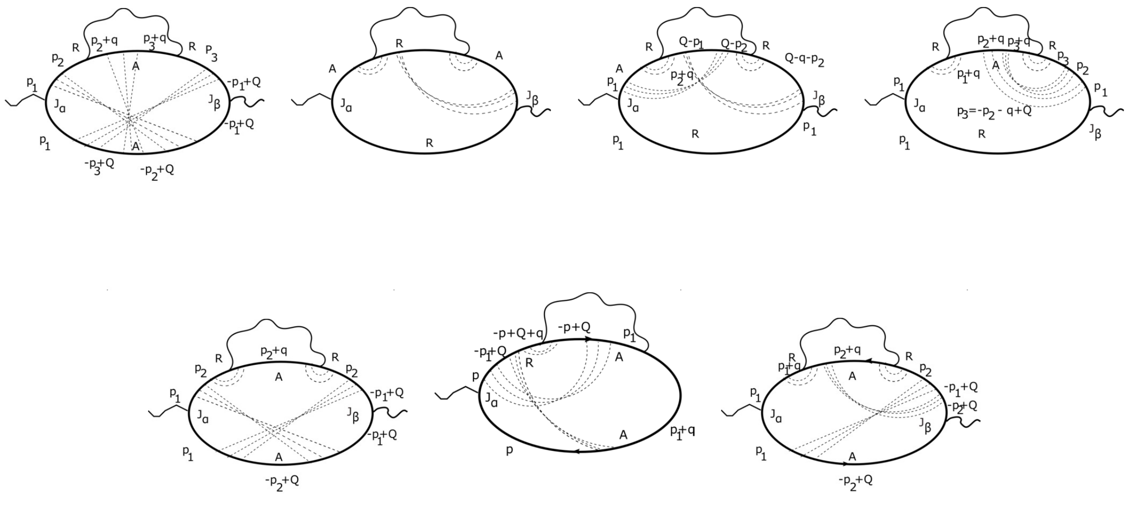

Fig. S1: The diagrams that contribute to the cross-term correction to conductance, σ_{CWAL}

.

X. SUPPLEMENTARY FIGURE S2

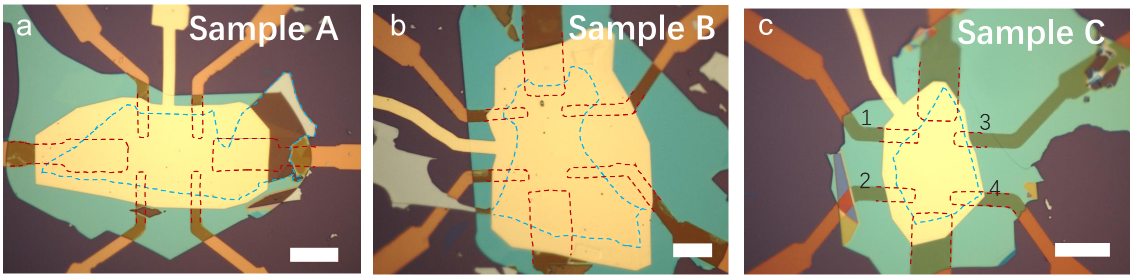

Fig. S2: Optical images of three dual-gated BSTS devices (samples A, B and C). All scale bars denote 15 μm.

XI. SUPPLEMENTARY FIGURE S3

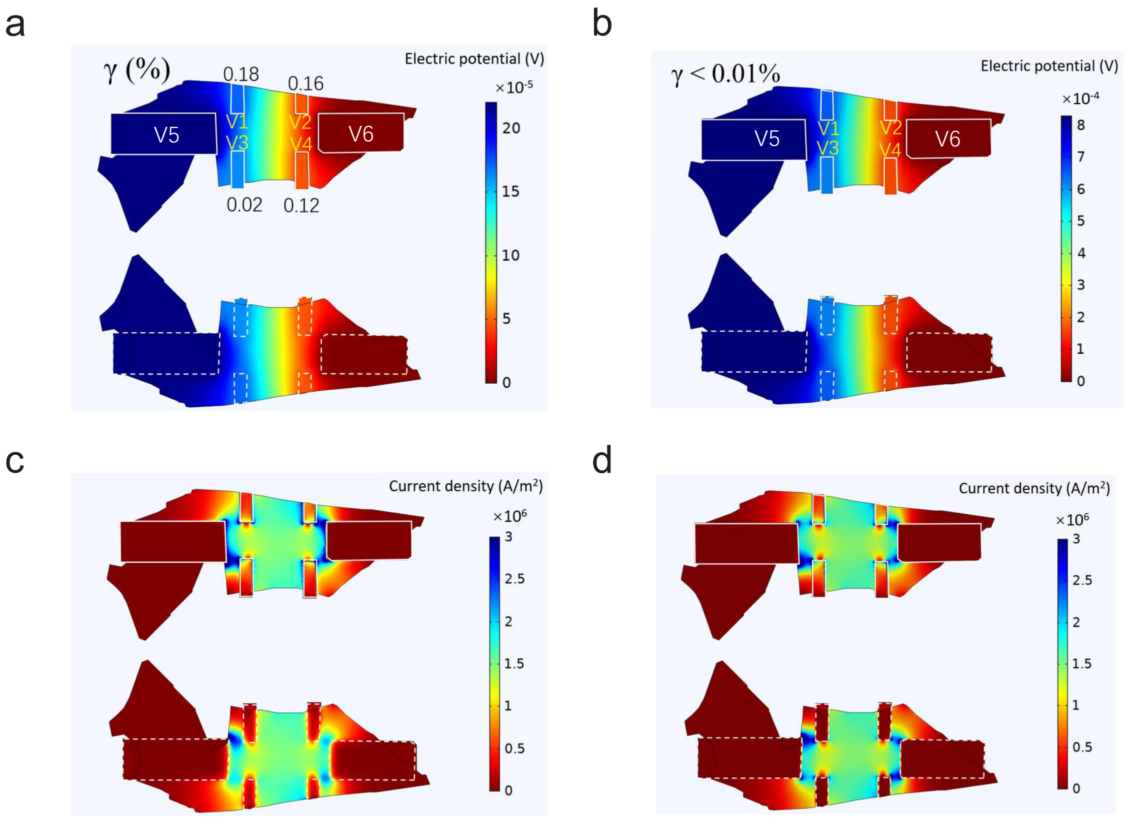

Fig. S3: Simulation of current density and electric field distributions in a BSTS device with finite element analysis. **a, b** Potential distribution on different surfaces with high bulk resistivity/low surface resistivity (**a**), and low bulk resistivity/high surface resistivity (**b**). **c, d** Current density distribution for high bulk resistivity (**c**) and low bulk resistivity (**d**). A source-drain current of $0.1\,\mu$A is used in the simulation.

XII. SUPPLEMENTARY FIGURE S4

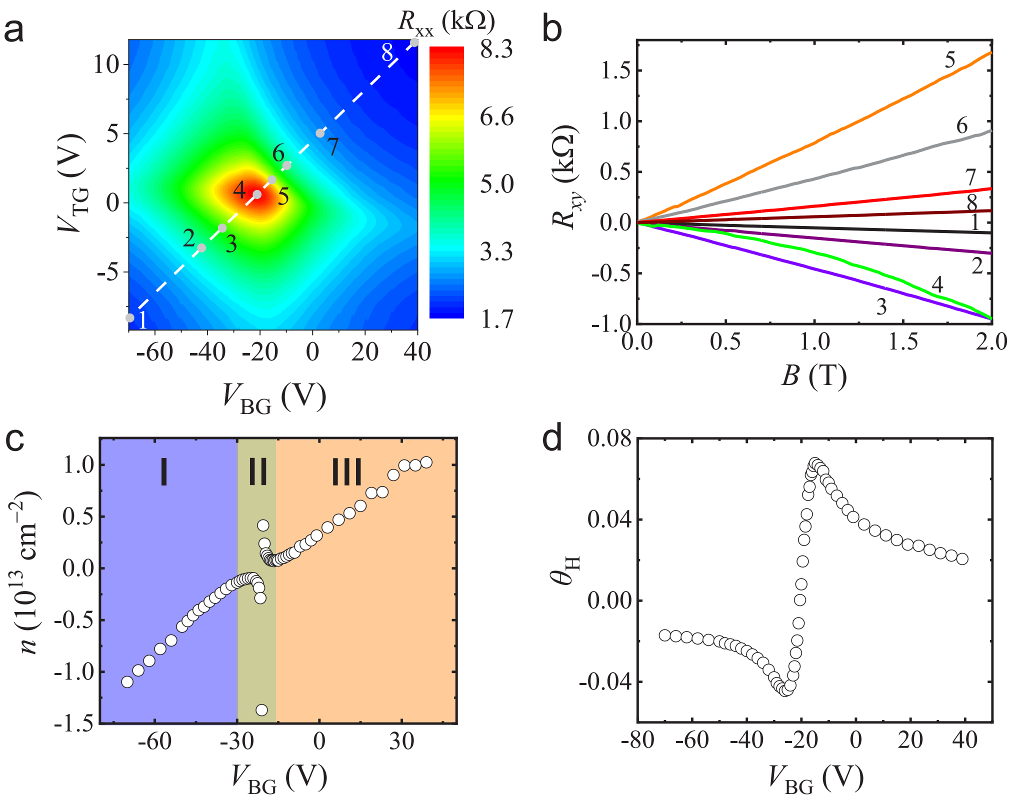

Fig. S4: Additional characterization of the basic transport properties of sample A at $T = 1.6\,\mathrm{K}$. **a** Sheet resistance per square plotted as a function of the top and bottom gate-voltages at zero magnetic field. **b** Hall effect curves for eight gate-voltage sets (points 1-8, marked in panel **a**). **c,d** Dependence of the nominal carrier density (**c**) and and the Hall angle (**d**) on the back-gate voltage along the symmetric gating line. The former is given by $n_{s,H} = -1/eR_H$, where R_H is the measured raw Hall coefficient without taking the geometrical correction factor f_H into account. The data shown here is complementary to Fig. 1 in the main body of this paper.

XIII. SUPPLEMENTARY FIGURE S5

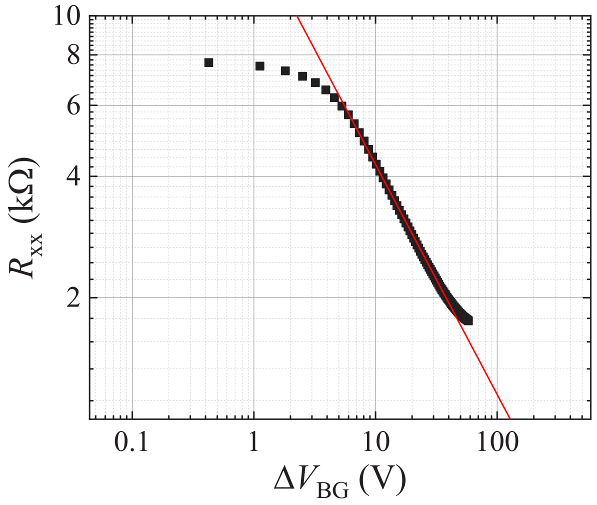

Fig. S5: Sheet resistance per square plotted as a function of $\Delta V_{\rm BG}$, which is the defined as the difference in the back-gate voltage with respect to the charge neutral point. The electron density is proportional to $\Delta V_{\rm BG}$ if the sample is not in the ambipolar regime. The data was taken at $T = 1.6\,$K and $B = 0$. The red line is a linear fit in the log-log scale.

XIV. SUPPLEMENTARY FIGURE S6

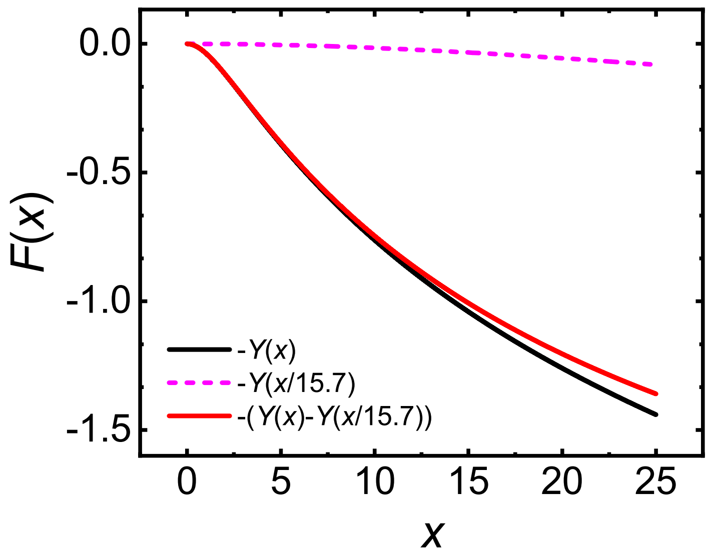

Fig. S6: Comparison of two approximate forms of the Maki-Thompson correction to magnetoconductance. The black solid line represents the function $F(x) = -Y(x)$, where $Y(x) = \psi(1/2 + 1/x) + \ln x$. The red solid line plots the function $F(x) = -\{Y(x) - Y[x/(2\pi B_T/B_\varphi)]\}$, with $B_T/B_\varphi = 2.5$ (close to the value for point C, near the charge neutral point). The former function is valid at $B \ll 2\pi B_T$, and it differs from the latter by a quadratic function, plotted as the upper pink curve (dashed).

XV. SUPPLEMENTARY FIGURE S7

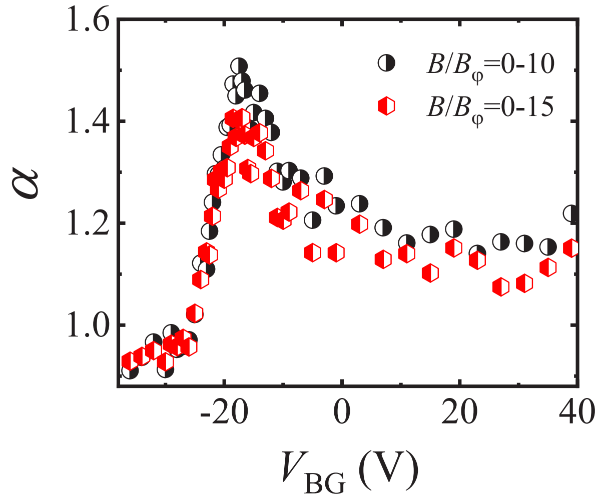

Fig. S7: Gate-voltage dependence of prefactor α at $T = 1.6\,\mathrm{K}$. The α values were extracted with the HLN fits of the MC data in a magnetic field range of $B = $ 0-10B_φ or 0-15B_φ.

XVI. SUPPLEMENTARY FIGURE S8

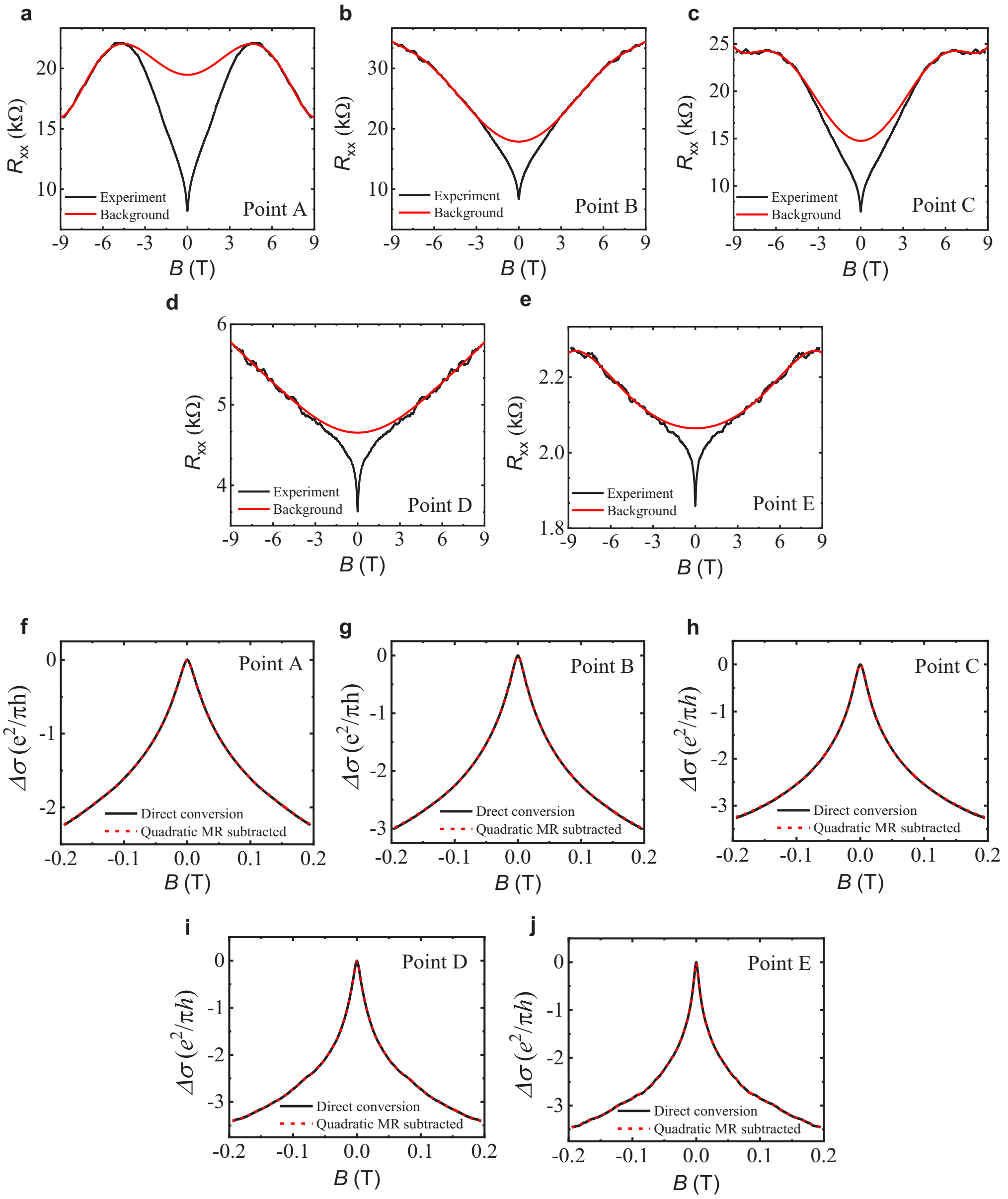

Fig. S8: Negligible influence of low field quadratic MR background on the HLN analysis of MC data of sample A. **a-e** Even-power polynomial fits of the MR data at $B = 4$-$9\,\mathrm{T}$ for points A-E. **f-j** Low field MC curves before (solid black) and after (dashed red) the quadratic background is subtracted. The bottom and top gate-voltages corresponding to points A-E can be found in Figs. 2 and 1 in the main body of this paper.

XVII. SUPPLEMENTARY FIGURE S9

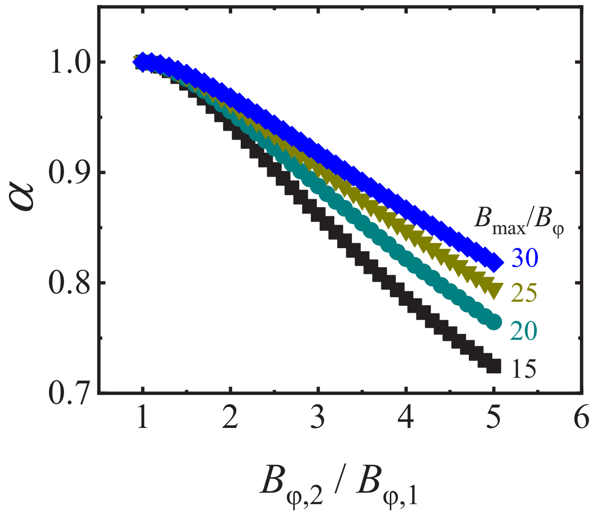

Fig. S9: Influence of unequal dephasing fields (lengths) on the top and bottom surfaces on the α value extracted with the HLN fit. Results of four different fitting ranges are included, namely B/B_φ = 0-15, 0-20, 0-25, and 0-30.

XVIII. SUPPLEMENTARY FIGURE S10

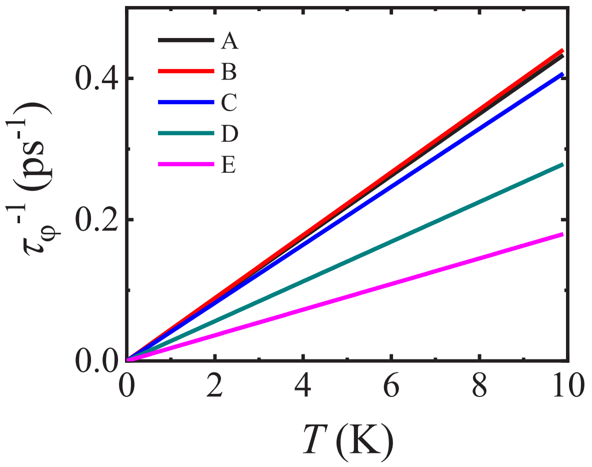

Fig. S10: Theoretical values of dephasing rate (τ_φ^{-1}) obtained by solving Eq. S70 self-consistently for the five gate-voltages sets (Points A-E in Fig. 2 in the main body of this paper).

XIX. SUPPLEMENTARY FIGURE S11

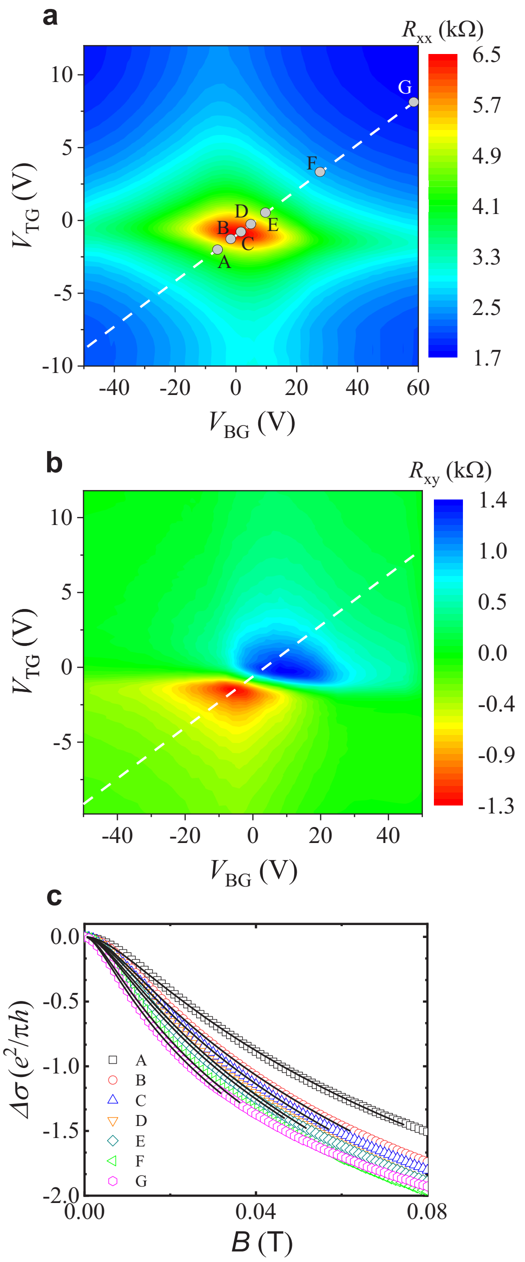

Fig. S11: Magneto-transport measurement results of sample B at $T = 1.6\,\mathrm{K}$. **a** Sheet resistance per square as a function of the top and bottom gate voltages at zero magnetic field. **b** Hall resistance at $B = 1\,\mathrm{T}$ as a function of V_{TG} and V_{BG}. **c** MC curves (symbols) and their fits to the HLN equation (lines) for seven gate-voltage sets, marked as points A-G in panel **a**.

XX. SUPPLEMENTARY FIGURE S12

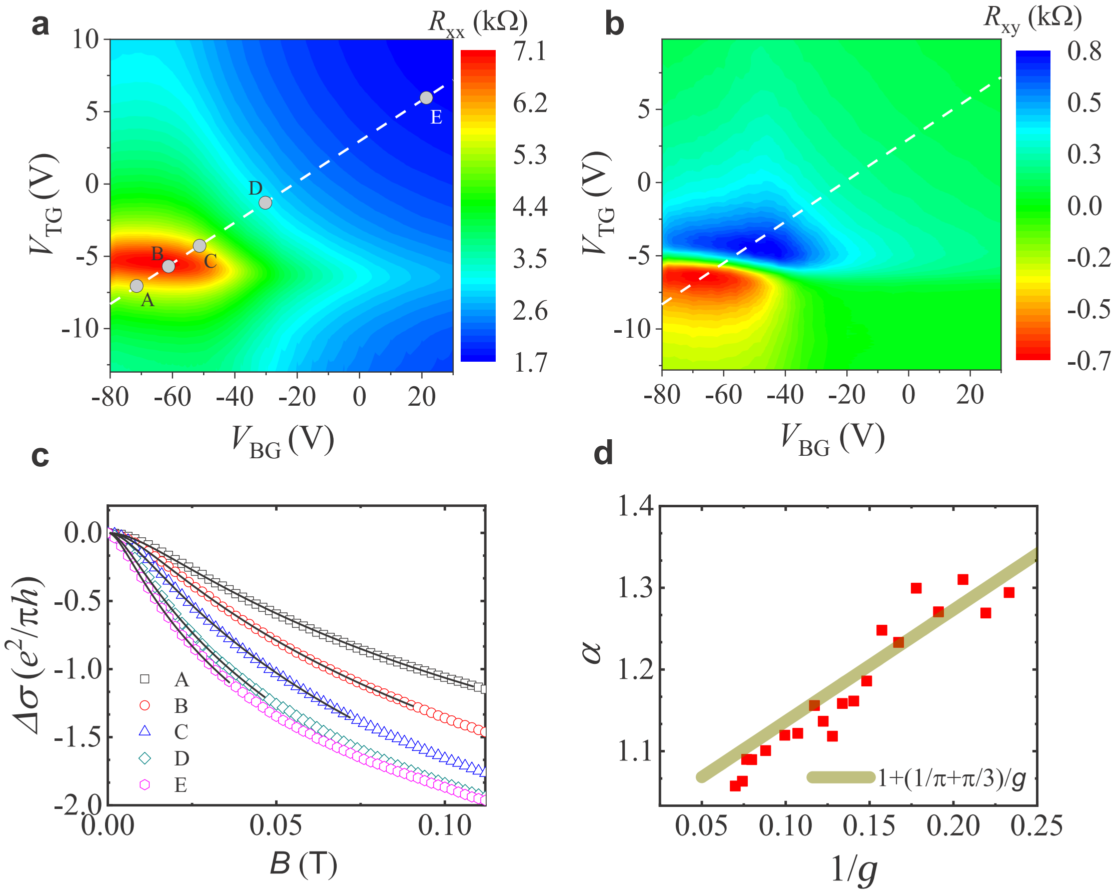

Fig. S12: Magneto-transport measurement results of sample C at $T = 1.6\,\mathrm{K}$. **a** Sheet resistance per square as a function of the top and bottom gate voltages at zero magnetic field. **b** Hall resistance at $B = 1\,\mathrm{T}$ vs. V_{BG} and V_{TG}. **c** MC curves (symbols) and their fits to the HLN equation (lines) for five gate-voltage sets, marked as points A-E in panel **a**. **d** Prefactor α, extracted from the HLN fit, plotted as a function of $1/g$. The experimental values (red sqaures) are compared with those given by Eq. 3 in the main text (yellow line).

XXI. SUPPLEMENTARY FIGURE S13

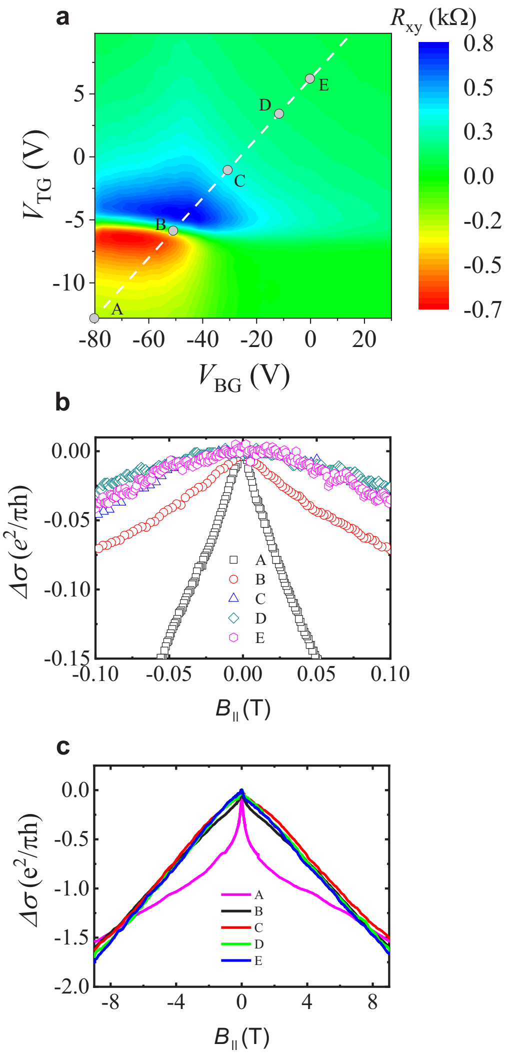

Fig. S13: Magnetoconductance data taken in parallel magnetic fields for sample C. **a** Hall resistance vs. $V_{\rm BG}$ and $V_{\rm TG}$ taken at $T = 1.6\,$K and in a perpendicular field of 1 T. **b, c** Parallel field MC curves for five gate-voltage sets (marked as points A-E in panel **a**). The low field quadratic MC curves (panel **b**)suggest a transport process dominated by the surface states (points C, D and E, with the Fermi level near CNP or above), whereas the deviation from the quadratic form at points A and B suggests the participation of the bulk states. The quasilinear MC at high magnetic fields ($B > 2\,$T, panel **c**) remains to be understood.

Supplementary References

S1 S. Hikami, A. I. Larkin, and Y. Nagaoka, Spin-Orbit Interaction and Magnetoresistance in the Two Dimensional Random System. *Prog. Theor. Phys.* **63**, 707-710 (1980).

S2 S. Hikami, Anderson localization in a nonlinear σ model representation. *Phys. Rev. B* **24**, 2671 (1981).

S3 E. Brezin, S. Hikami, and J. Zinn-Justin, Generalized Non-Linear Sigma-Models with Gauge-Invariance. *Nucl. Phys. B* **165**, 528 (1980).

S4 F. Wegner, Disordered Electronic System as a Model of Interacting Matrices. *Phys. Rep.* **67**, 15 (1980).

S5 Minkov, G. M., Germanenko, A. V. and Gornyi, I. V. Magnetoresistance and dephasing in a two-dimensional electron gas at intermediate conductances. *Phys. Rev. B* **70**, 245423 (2004).

S6 A. M. Finkel'stein, Electron liquid in disordered conductors, Soviet Scientific Reviews, Vol. 14, edited by I. M. Khalatnikov (Harwood Academic Publishers, 1990).

S7 B. L.Altshuler and A. G. Aronov, in Electron–Electron Interactions in Disordered Systems, Modern Problems in Condensed Matter Sciences, Vol. 10, edited by A. L. Efros and M. Pollak (publisher Elsevier, 1985).

S8 I. Aleiner, B. Altshuler, and M. Gershenson, *Waves in Random Media* **9**, 201 (1999).

S9 L. Golub, I. Gornyi, and V. Y. Kachorovskii, Weak antilocalization in two-dimensional systems with large Rashba splitting. *Phys. Rev. B* **93**, 245306 (2016).

S10 I. Burmistrov, I. Gornyi, and A. Mirlin, *Phys. Rev. B* **92**, 014506 (2015).

S11 A. I. Larkin, Reluctance of Two-Dimensional Systems. *JETP Lett.* **31**, 239 (1980).

S12 K. Maki, The Critical Fluctuation of the Order Parameter in Type-II Superconductors. *Prog. Prog. Theor. Phys.* **39**, 897 (1968).

S13 R. S. Thompson, Microwave, Flux Flow, and Fluctuation Resistance of Dirty Type-II Superconductors. *Phys. Rev. B* **1**, 327 (1970).

S14 F. Gao and Y. Li, Influence of Device Geometry on Transport Properties of Topological Insulator Microflakes. *Chin. Phys. Lett.* **38**, 117302 (2021).

S15 A. C. Neto, F. Guinea, N. M. Peres, K. S. Novoselov and A. K. Geim, The electronic properties of graphene. *Rev. Mod. Phys.* **81**, 109 (2009).

S16 D. X. Qu, Y. S. Hor, J. Xiong, R. J. Cava, and N. P. Ong, Quantum oscillations and Hall anomaly of surface states. in the topological insulator Bi_2Te_3 Science **329**, 821 (2010).

S17 G. H. Zhang, H. J. Qin, J. Chen, X. Y. He, Y. Q. Li, and K. H. Wu, Growth of topological insulator Bi2Se3 thin films on $SrTiO_3$ with large gate-voltage-tunable chemical potential. Adv. Func. Mater. **21**, 2351 (2011).

S18 Y. Kumar, R. Sultana, and V. P. S. Awana, Comprehensive analysis for the high field magneto-conductivity of Bi_2Te_3 single crystal. Physica B **609**, 412759 (2021).

S19 Y. Xu, I. Miotkowski, C. Liu, J. F. Tian, H. Nam5, N. Alidoust, J. N. Hu, C.-K. Shih, M. Z. Hasan, and Y. P. Chen, Observation of topological surface state quantum Hall effect in an intrinsic three-dimensional topological insulator. Nat. Phys. **10**, 956 (2014).

S20 K. B. Han, S. K. Chong, A. O. Oliynyk, A. Nagaoka, S. Petryk, M. A. Scarpulla, V. V. Deshpande, and T. D. Sparks, Enhancement in surface mobility and quantum transport of $Bi_{2-x}Sb_xTe_{3-y}Se_y$ topological insulator by controlling the crystal growth conditions. Sci. Rep. **8**, 17290 (2018).

---

* Electronic address: Dmitri.Gutman@biu.ac.il

† Electronic address: yqli@iphy.ac.cn
